# Developing organs‐on‐chips for biomedical applications

**DOI:** 10.1002/SMMD.20240009

**Published:** 2024-05-21

**Authors:** Lingyu Sun, Hanxu Chen, Dongyu Xu, Rui Liu, Yuanjin Zhao

**Affiliations:** ^1^ Department of Rheumatology and Immunology Nanjing Drum Tower Hospital School of Biological Science and Medical Engineering Southeast University Nanjing China; ^2^ Mechanobiology Institute National University of Singapore Singapore Singapore

**Keywords:** biosensing, cell culture, drug screening, microfluidics, organs‐on‐chips, tissue engineering

## Abstract

In recent years, organs‐on‐chips have been arousing great interest for their bionic and stable construction of crucial human organs in vitro. Compared with traditional animal models and two‐dimensional cell models, organs‐on‐chips could not only overcome the limitations of species difference and poor predict ability but also be capable of reappearing the complex cell‐cell interaction, tissue interface, biofluid and other physiological conditions of humans. Therefore, organs‐on‐chips have been regarded as promising and powerful tools in diverse fields such as biology, chemistry, medicine and so on. In this perspective, we present a review of organs‐on‐chips for biomedical applications. After introducing the key elements and manufacturing craft of organs‐on‐chips, we intend to review their cut‐edging applications in biomedical fields, incorporating biological analysis, drug development, robotics and so on. Finally, the emphasis is focused on the perspectives of organs‐on‐chips.

## Graphical abstract

Organs‐on‐chips can reproduce the crucial structures and functions of human organs, thus providing powerful tools for revealing the physiological mechanisms and drug testing. This perspective discusses the advanced fabrication techniques of organs‐on‐chips and their biomedical applications such as toxicity evaluation and robotics. The emphasis is focused on the remaining challenges and future perspectives of organs‐on‐chips.

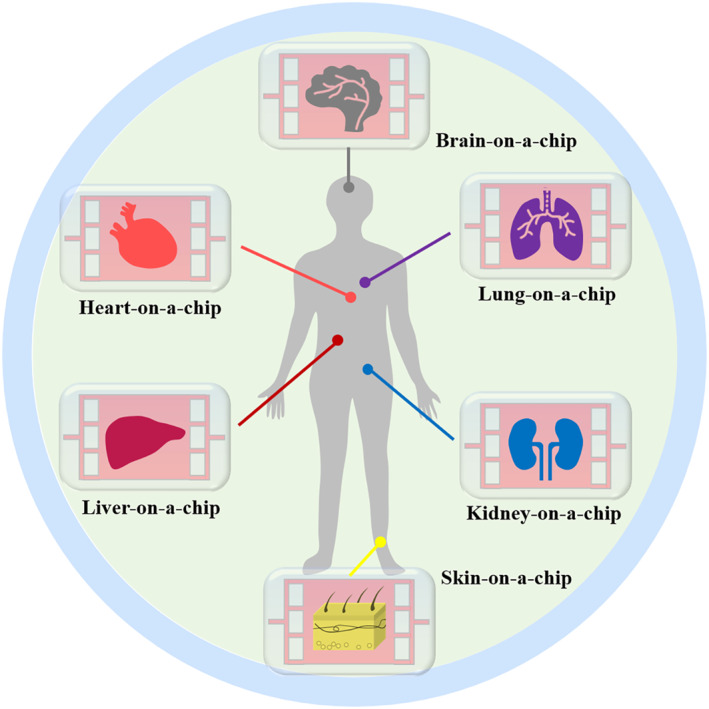

**Key points**

- The crucial elements for the fabrication of functional organs‐on‐chips are overviewed.
- The cut‐edging biomedical applications of organs‐on‐chips are introduced.
- The prospects and challenges of organs‐on‐chips are discussed.

## 1 INTRODUCTION

Organs‐on‐chips aim to construct a physiological microsystem for simulating the crucial structures and functions of human organs.^1^, ^2^, ^3^ Benefitting from the progress of material science and cell biology, various organs‐on‐chips have been proposed, such as heart‐on‐a‐chip,^4^, ^5^ lung‐on‐a‐chip,^6^ liver‐on‐a‐chip,^7^ brain‐on‐a‐chip,^8^ gut‐on‐a‐chip^9^ and so on.^10^, ^11^, ^12^, ^13^ These biomimetic systems demonstrate unparalleled superiority in providing biofluidic conditions, facilitating intercellular interactions, reconstructing biological structures and functions, etc. More importantly, these different types of organs‐on‐chips could be effectively combined to simulate the complex interaction among organs,^14^, ^15^ thus enhancing the accuracy of predicting the possible reactions of human physiological system to different stimulations. When integrated with advanced bioimaging and detection strategies, the weak cellular behaviors in organs‐on‐chips could be efficiently magnified and monitored in real time, which contributes to revealing the underlying biological or pathological mechanisms of organisms.^16^, ^17^, ^18^, ^19^, ^20^ Based on these achievements, organs‐on‐chips have emerged as promising tools to replace conventional two‐dimensional (2D) cell or animal models for biological research, drug screening, vaccine development, personalized medicine, etc.^21^, ^22^, ^23^

In this paper, we introduce the progress of organs‐on‐chips for biomedical applications and propose the possible technological advancements in the future. After introducing the common manufacture techniques such as soft lithography, laser etching and bioprinting, we would present some typical examples of organs‐on‐chips like lung‐on‐a‐chip and multi‐organs‐on‐chips that are arousing great interest in recent years. Emphasis would be given to the advanced applications of organs‐on‐chips in biomedical fields incorporating biological analysis, drug development, robotics and so on. Finally, the remaining challenges and perspectives of organs‐on‐chips are also discussed.

## 2 FABRICATION OF ORGANS‐ON‐CHIPS

**FIGURE 1:**
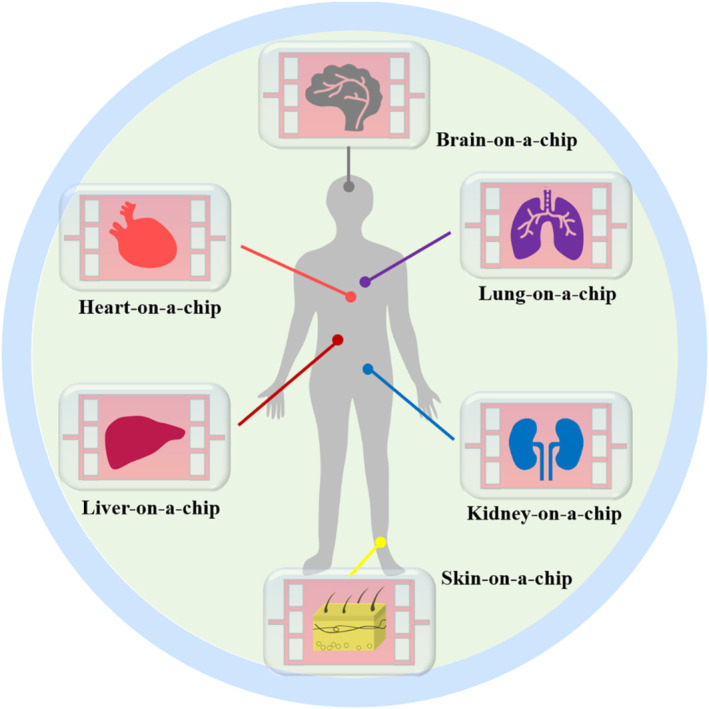
Schematic diagram showing the construction of organs‐on‐chips.

**FIGURE 2:**
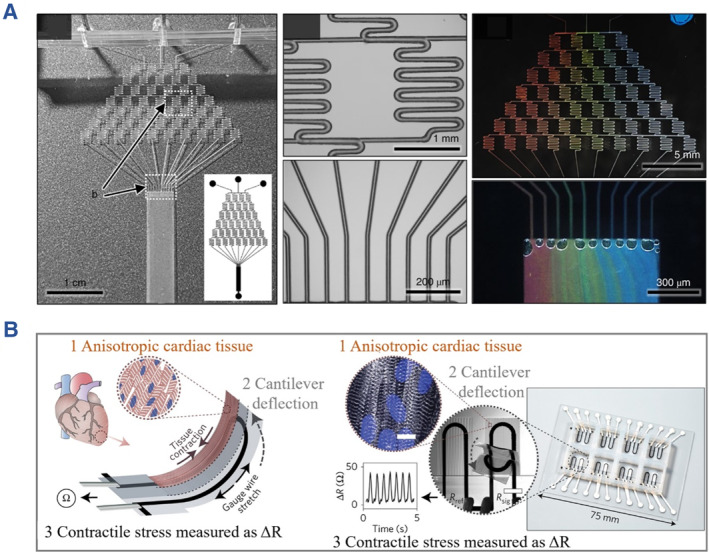
(A) Gradient microfluidic chips fabricated from successive laser pyrolysis technique. Reproduced with permission.^32^ Copyright 2021, The Authors, published by Springer Nature. (B) A fully 3D‐printed heart‐on‐a‐chip with integrated strain sensor. Reproduced with permission.^33^ Copyright 2017, Springer Nature.

Because traditional in vitro models are difficult to predict actual human responses, great interest has been focused on developing functional organs‐on‐chips as alternatives (Figure 1). During the fabrication of organs‐on‐chips, the key considerations include microfluidic chips, stimulation element, biosensing unit, and biological components.^24^, ^25^ Numerous strategies have been developed to manufacture microfluidic chips with elaborate chamber and channel designs, such as photolithography,^26^ molding,^27^ laser etching,^28^ etc.^29^, ^30^, ^31^ These strategies possess their own merits and demerits, and efforts are still devoted to the improvement of existing strategies or the development of novel ones. To overcome the limitations (e.g. time‐consuming and expensive) of frequently‐used photolithography and molding methods, Shin et al. employed a laser pyrolysis technique to directly process polydimethylsiloxane (PDMS) chips with elaborate structures, as shown in Figure 2A.^32^ However, the components of the chips are usually prepared, followed by an assembly, bonding and encapsulation process to form an independent and controllable microenvironment. Recently, much effort has been focused on constructing integrated microfluidic chips by one‐step methods based on techniques like three‐dimensional (3D) printing.^34^, ^35^, ^36^ In this case, the precision and complexity of chips are further enhanced, while the stimulation and detection units could even be integrated into the chips during the fabrication process. When the stimulations are implemented by electrodes or chemicals, the real‐time response of cells could be monitored and reflected by the sensing unit. As an example, Lind et al. described a novel 3D‐printed instrumented cardiac microphysiological device with embedded strain sensor and output unit, which provided a non‐invasive and simplified detection for contractile stress of cardiomyocytes (Figure 2B).^33^

**FIGURE 3:**
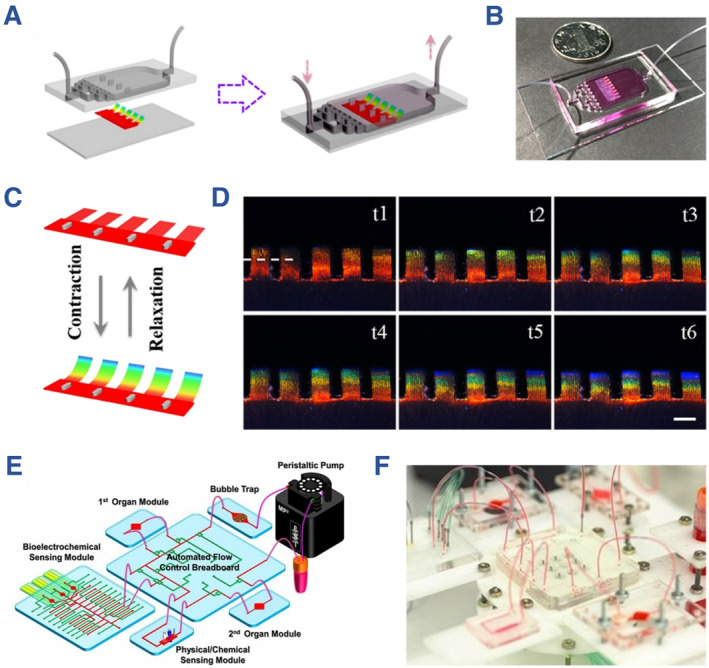
(A, B) Schematic diagram and optical image of the structural color‐hydrogel integrated microfluidic chip. (C, D) Scheme and photographs showing the color change under actuation of cardiomyocytes. (A–D) Reproduced with permission.^45^ Copyright 2018, The Authors, published by the American Association for the Advancement of Science. (E, F) Scheme and optical photograph of the organs‐on‐a‐chip system integrated with multiple sensors, respectively. (E, F) Reproduced with permission.^46^ Copyright 2017, National Academy of Sciences.

Among various components of organs‐on‐chips, the biosensing unit plays an important role in visualization of microscopic cellular activity. An ideal biosensing unit ought to possess high sensitivity, miniaturized volume and biosecurity. Traditional detection strategies containing impedance sensors,^37^, ^38^ mechanical sensors,^39^ metabolite sensors,^40^ etc.,^41^, ^42^, ^43^ have realized the highly sensitive detection of morphological, electrical and mechanical cellular performances in chips. Aleman et al. introduced an electrochemical affinity‐based biosensor for in situ and simultaneous detection of multiplexed biomarkers in organs‐on‐chips.^44^ The derived microfluidic chip was designed into three layers, including valve top layer, membrane middle layer and bottom flow layer embedded with electrode. To simplify the detection process, Fu and his co‐workers introduced cardiomyocyte‐engineered structural color hydrogels into the microfluidic system and thus achieved visualized heart‐on‐chips, as shown in Figure 3A–D.^45^ After that, Shang et al. and Xu et al. also verified the feasibility and practicability of structural color‐based organs‐on‐chips for drug screening and cardiotoxicity screening, respectively.^47^, ^48^ Although the structural color‐based optical sensors simplify the sensing process, their stability and sensitivity still need improvement. For realizing dynamic and precise evaluation of cellular microenvironment, Zhang et al. developed a class of organ‐on‐chips integrated with multiple classifications of sensors including physical, electrochemical and optical ones (Figure 3E,F).^46^ Such multi‐sensor integrated platform was suitable for in situ monitoring of biophysical and biochemical parameters of organ models in an accurate, automated, noninvasive and real‐time manner, thus expanding the value of organs‐on‐chips for long‐term cell culture.

In addition to the nonliving parts, cell culture is also a critical issue worthy to be explored, where culture mode (2D or 3D), cell sources (including immortalized cells, primary cells and stem cells) and cell types (single cell culture or co‐culture) should be taken into consideration. To be specific, the culture mode should be chosen according to the actual cellular morphology in vivo. For instance, 2D cell culture is suitable for 2D tissues such as alveolar epithelial cells, while 3D culture mode applies to solid organs like liver, heart and even tumors. With regard to cell sources, stem cells, especially induced pluripotent stem cells (iPSCs), are potential candidates for the construction of next generation of organs‐on‐chips because of their advantages in totipotency, credibility and without ethical issues compared to immortalized cells and primary cells.^49^, ^50^, ^51^, ^52^ In particular, the iPSCs‐based organs‐on‐chips could be tailored according to individual patients thanks to the advance in gene editing technique. To better simulate the in vivo microenvironment, the cells are usually induced into specific spatial arrangement and applied with electrical or chemical stimulations for promoting maturation and differentiation.^53^, ^54^ In addition, different cell lines could be co‐cultured in an integrated microfluidic chip to investigate the intercellular interactions and organ interactions, thus laying the foundation of subsequent biological/pathology mechanism study and drug development.^55^

**FIGURE 4:**
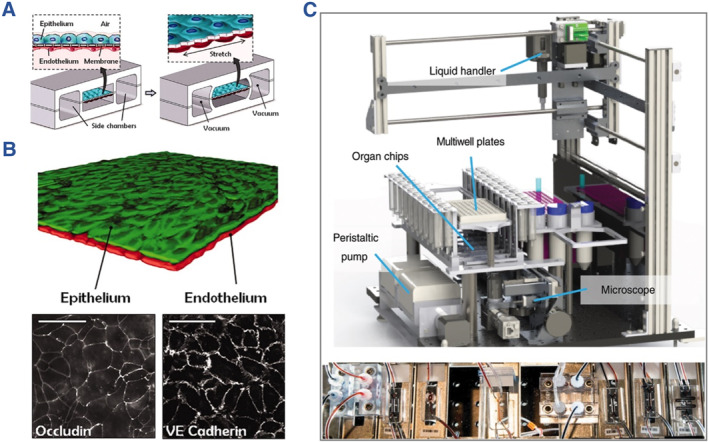
(A) Schematic diagram showing the construction of breathable lung‐on‐a‐chip with alveolar‐capillary interface. (B) Cellular staining results showing the monolayer formation of epithelial and endothelial cells distributed on the opposite interface of the PDMS membrane. (A, B) Reproduced with permission.^56^ Copyright 2010, the American Association for the Advancement of Science. (C) Photographs of the integrated instrument for multi‐organ cultivation and monitoring. Reproduced with permission.^57^ Copyright 2020, The Authors, published by Springer Nature.

Based on these advanced techniques, microfluidic chips cultured with different organ analogues including heart, lung, brain, kidney, liver and vessel have been presented and display certain biological characteristics for in vitro investigation.^4^, ^5^, ^6^, ^7^, ^8^, ^9^, ^10^, ^11^, ^12^, ^13^ A milestone in the organs‐on‐chips history is the lung‐on‐a‐chip proposed by Huh et al. in 2010, which realized the reconstitution of human alveolar‐capillary interface, as shown in Figure 4A,B.^56^ Since then, the area of organs‐on‐chips has entered a rapid development period, when the precision, bionic performance, throughput and lifetime of the fabricated organs‐on‐chips have been substantially improved. With the achievements, microfluidic chips coupled with multiple organs have emerged, which greatly promote the progress of organs‐on‐chips towards real human‐on‐a‐chip.^58^ As an example, Novak et al. put forward an integrated instrument that linked with ten organ modules by shared vascularized channel (Figure 4C).^57^ This design could not only greatly mimic the physiological transport of small molecules among organs, but also realize long‐term culture of organs up to 3 weeks. These biomimetic platforms preliminarily proved their values in laboratory investigation. Nowadays, researchers are devoting themselves to cooperating with companies to promote the progress of organs‐on‐chips from the laboratorial stage into practical applications.

## 3 ADVANCED APPLICATIONS IN BIOMEDICAL ENGINEERING

### 3.1 Organs‐on‐chips for biological analysis

**FIGURE 5:**
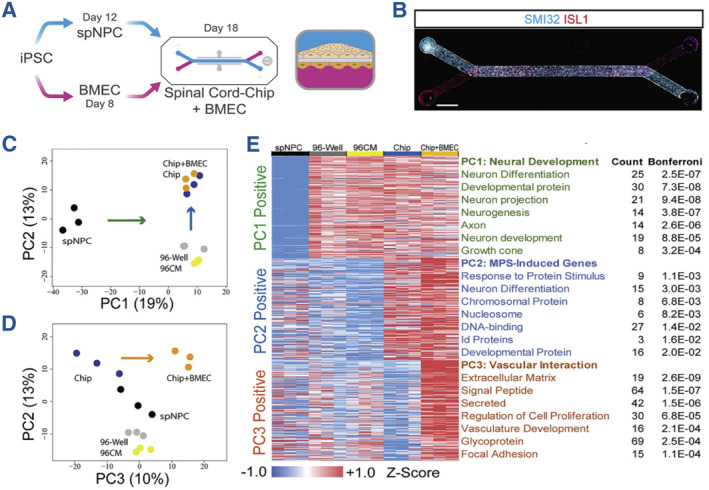
(A) Diagram of the construction of the microfluidic chip co‐cultured with spinal neural cells and brain microvascular endothelial cells. (B) Immunostaining results showing the biomarker expression of these two cells (SMI32 for neural cells and ISL1 for vascular cells). (C–E) Gene pathway analysis of neuronal development and vascular interaction under different conditions. (A–E) Reproduced with permission.^63^ Copyright 2018, The Authors, published by Elsevier.

Benefitting from the advances of microfluidics, cell biology, and machine learning fields, organs‐on‐chips have evolved into powerful tools for biomedical engineering fields such as biological analysis, drug development and toxicity evaluation, robotics, etc.^21^, ^22^, ^23^, ^59^ In these systems, the biological behaviors of cells including the cellular proliferation, morphology, activity, metabolism and so on could be accurately monitored by the integrated sensing unit in the chip for further analysis. More importantly, researchers could even regulate cellular behaviors by applying different external stimulations. With these efforts, organs‐on‐chip systems have demonstrated value in reappearing crucial structural and functional features of human organs. Therefore, organs‐on‐chips are applicable to investigate the underlying biological and pathogenesis mechanisms of specific organ/tissue models.^60^, ^61^, ^62^ For example, Sances et al. took advantage of the microengineered organ‐on‐a‐chip system for the co‐culture of stem‐cell derived neural and vascular cells, as shown in Figure 5A,B. They found that the chip system promoted neuron development than 96‐well plate, while the co‐culture mode facilitated the formation of vascular‐neural interaction and activation of specific gene (Figures 5C–E).^63^ In addition to the research of single organ, the microfluidic platform also shows potential in studying the interaction among different organs, which may contribute to the further understanding of complicated mechanisms of human body.^64^, ^65^ Moreover, organs‐on‐chips have also been extensively applied for revealing the pathophysiological phenomenon of diseases such as respiratory ones.^66^ In general, the advances in fields like cell biology promote the emergence of organs‐on‐chips, which in turn promotes the in‐depth investigation and understanding of these disciplines.

### 3.2 Organs‐on‐chips for drug development

**FIGURE 6:**
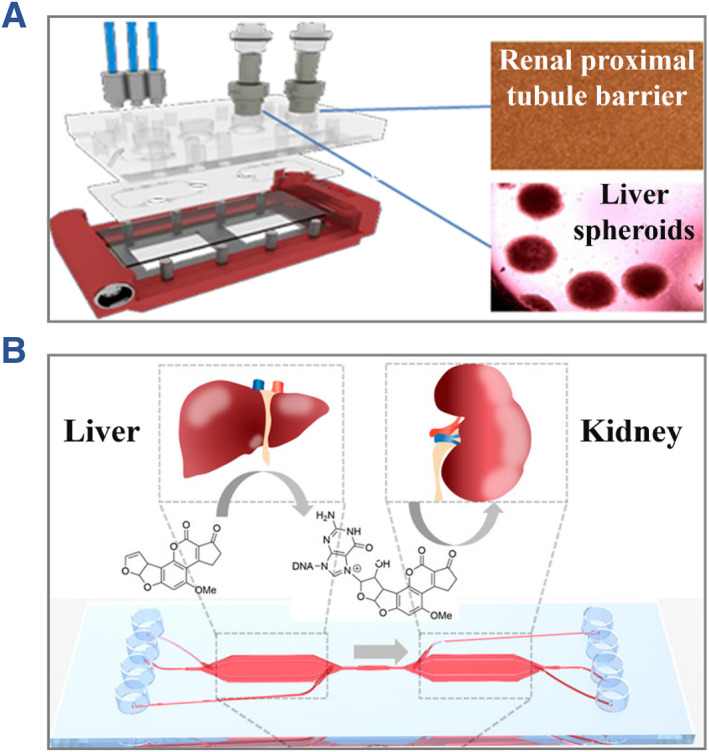
(A) Schematic diagram showing the live‐proximal tubule coupled organ‐on‐a‐chip. Reproduced under terms of the CC‐BY license.^75^ Copyright 2020, The Authors, published by Springer Nature. (B) A simplified liver‐kidney chip for the evaluation of environmental toxins. Reproduced with permission.^76^ Copyright 2018, American Chemical Society.

Drug development plays a vital role in preventing, treating and curing diseases, which has aroused widespread interest of the society.^67^ Considerable costs and time are devoted to the synthesis and evaluation of new drugs each year. Since traditional animal models failed to accurately predict the drug effects on human, great effort has been focused on developing more reliable, accurate and effective models for evaluating the metabolism and potential toxicity of drug candidates.^68^, ^69^, ^70^ In recent years, organs‐on‐chips have shown demonstrated value in promoting the drug development progress, owing to their advantages of reproducing physiological conditions, high‐throughput property, low consumption and so on.^71^, ^72^, ^73^, ^74^ In these systems, the cellular behaviors in response to different stimulus could be magnified and recorded, which is difficult to realize by traditional methods. Because liver and kidney are two main target organs of toxic compounds, Lin et al. constructed a microfluidic chip co‐cultured with liver tissue and renal proximal tubule barriers for drug testing (Figure 6A).^75^ The results revealed the effect of rifampicin on accelerating the metabolism and inhibiting the toxicity of cyclosporine A. Similarly, Theobald et al. developed a simplified liver‐kidney chip and utilized the system for environmental toxins evaluation, as shown in Figure 6B.^76^ They found that the Aflatoxin B1 showed much higher toxicity on hepatic cells than kidney cells, attributed to the high expression of CYP in hepatic cells which would convert Aflatoxin B1 into toxic products. For better modeling of human physiology, Ronaldson‐Bouchard et al. proposed a multi‐organ chip of heart, liver, bone and skin niches that connected by circulating vascular flow, and verified the practicability of such platform in predicting the toxicity of doxorubicin.^77^ These instances proved the potential of organs‐on‐chips to predict the possible effect of human organs in response to diverse chemicals.

It is worth mentioning that the microfluidic chips are capable of forming drug gradients through bifurcated design of injection channels, thus adapting to the demand in high‐throughput drug screening.^78^, ^79^ For instance, Chen et al. established a structural‐color‐microfiber integrated heart‐on‐a‐chip and demonstrated its simultaneous screening of five concentrations of isoproterenol solution on the contraction of cardiomyocytes.^80^ By adding the numbers of chambers and microchannels, the organs‐on‐chips are applicable for high‐throughput cell culture and drug evaluation. Tan et al. proposed a 96 microfluidic array equipped with a recirculation pumping system to meet the high‐throughput requirements in large‐scale drug screening.^81^ By adjusting the configurations of microfluidic chips, organs‐on‐chips could flexibly meet different demands for different application scenarios.

**FIGURE 7:**
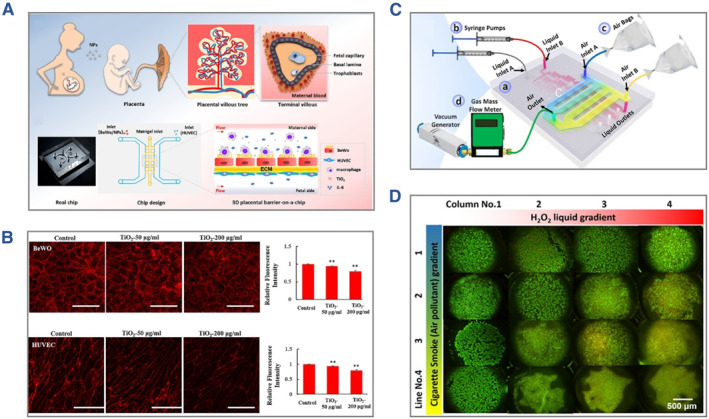
(A) Diagram of the construction of placental model for evaluation of titanium dioxide nanoparticles. (B) Characterization of the integrity of placental barrier after the treatment of different concentrations of nanoparticles. (A, B) Reproduced with permission.^85^ Copyright 2019, Elsevier. (C) The scheme of the lung‐on‐a‐chip with liquid‐air dual‐gradient. (D) Fluorescent images showing the synergistic effect of H_2_O_2_ treatment and cigarette smoke exposure on cell viability. (C, D) Reproduced with permission.^86^ Copyright 2018, Elsevier.

Apart from the chemical compounds, pollutants such as nanoparticles, smoke and causative agents may also cause severe symptoms in the human body.^82^, ^83^, ^84^ Therefore, it is necessary to construct an evaluation strategy to predict the potential harm of pollutants and develop corresponding therapies. As an example, Yin et al. presented a 3D placental model and investigated its response to titanium dioxide nanoparticle exposure.^85^ The study showed that the nanoparticles would damage the integrity of placental barrier and immune function, as shown in Figure 7A,B. Liu et al. described a novel lung‐on‐a‐chip with liquid‐air dual‐gradient for evaluating the harmful effect of air pollutants (Figure 7C).^86^ As a typical pollutant model, cigarette smoke was then utilized to treat inflammatory cells induced by H_2_O_2_ and normal cells, respectively. The results suggested that inflammatory cells were more vulnerable to cigarette smoke (Figure 7D). Since the organs‐on‐chips are suitable for preclinical testing, they could serve as a bridge between scientific research and medical applications. In 2022, the FDA Modernization Act 2.0 eliminated the mandatory utilization of animal testing in drug development processes and promoted the application of novel models including organs/organoids‐on‐chips for preclinical testing models. Meanwhile, the enterprises related with organs‐on‐chips or organoids‐on‐chips entered into a booming period. With the continuous development of medical technology, the organs‐on‐chips may serve as core components to promote the development of minimized and integrated medical instruments in the future.

### 3.3 Organs‐on‐chips for robotics

**FIGURE 8:**
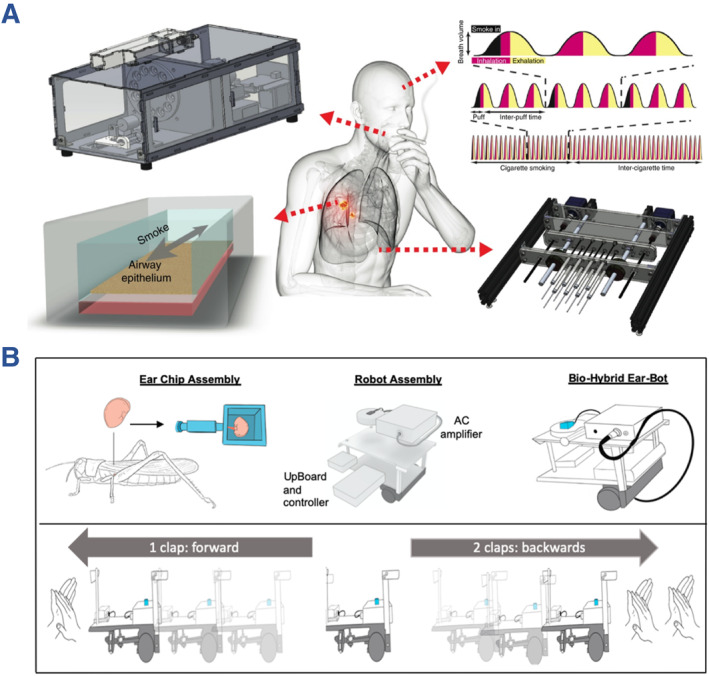
(A) Schematic diagram showing the components of the smoking robot. Reproduced with permission.^94^ Copyright 2020, The Authors, published by Springer Nature. (B) Fabrication of an ear‐on‐a‐chip and its controllable movement in response to external commands. Reproduced under terms of the CC‐BY license.^95^ Copyright 2021, The Authors, published by MDPI.

Robotics, especially soft and miniaturized ones, are gaining importance in biomedical fields such as drug delivery and surgery assistance in the human body.^87^, ^88^, ^89^, ^90^ Because organs‐on‐chips could reproduce physiological environments or construct disease models of humans, it is conceivable that organs‐on‐chips could become powerful tools for the testing of robotics.^91^, ^92^, ^93^ For example, Sun et al. fabricated a biohybrid soft robot‐integrated heart‐on‐a‐chip and demonstrated its practicability for drug screening.^79^ Apart from developing microrobots inside organs‐on‐chips, the whole chip could serve as functional modules of robots. Benam et al. developed a lung‐on‐a‐chip compatible smoking robot to study the effect of cigarette exposure on lung cells (Figure 8A).^94^ In addition, Fishel and co‐workers extracted the tympanic ear of insect and cultured it in a microfluidic chip, which could be further integrated with a robotic system, as shown in Figure 8B.^95^ The electrophysiological response of the insect ear to voice commands was monitored by electrodes, magnified by amplifier and then possessed for the control of robot moving. Although the application of organs‐on‐chips in robotic research is still in its infancy, organs‐on‐chips have shown the capacity of providing grounds for the emergence of next‐generation intelligent robotics and their applications in medicine.

## 4 CONCLUSION AND PERSPECTIVE

During the past decade, the organs‐on‐chips field has experienced an amazing evolution for their potential in replacing traditional cell and animal models, attributed to their advantages of miniaturization, integration, accuracy, bionic microenvironment, etc. Benefitting from the advancement of material science and biology, multifarious organ‐on‐a‐chip systems such as heart‐on‐a‐chip, lung‐on‐a‐chip, brain‐on‐a‐chip, etc., have sprung up. In recent years, researchers have attempted to integrate machine learning techniques with organs‐on‐chips to evaluate large amounts of data in a more effective and accurate manner. Based on these advances, organs‐on‐chips are playing an increasingly important role in medicine and healthcare. Although encouraging progress has been made, there are still several aspects worth considering to promote the commercial process and clinical transformation of organs‐on‐chips.

From the technical perspective, efforts should be devoted to the biomimetic construction of the organs‐on‐chips. Since the human body involves extremely complicated mechanisms and interactions, counting on the simplified organs‐on‐chips to reproduce the whole features of human organs is almost impossible. Considering that, the selection of local functions corresponding to diseases is instructive for the fabrication of next‐generation organs‐on‐chips. For this purpose, the biologists and medical researchers should cooperate to reveal the correspondence between diseases and biological functions of organs, and establish the target goals for different types of organs‐on‐chips.

After establishing definite targets, how to construct organs‐on‐chips including the cell sources and microfluidic chips also deserves profound discussion. With regard to cell sources, the primary cells extracted from living tissues are very limited and may even exist ethical issues. In contrast, iPSCs from somatic cell reprogramming have superiority in totipotency and indefinite self‐renewing ability, thus achieving great attention to serve as fundamentals for organ models.^96^, ^97^, ^98^ Further research has found that stem cell‐derived organoids are promising tools for investigating the development, homeostasis, and even diseases of human organs.^2^, ^60^ Instances of the integration of organoids and microfluidic systems have emerged and these integrated systems preliminarily demonstrated their value in accurate drug testing, disease modeling and so on.^99^, ^100^, ^101^, ^102^, ^103^ Despite the remarkable progresses, how to combine more organoids together for further improvement of their functions still requires the synergetic design and parameter optimization of biologists and engineers.

When it comes to the fabrication of microfluidic chips, the majority existing chips depend on the machining and encapsulation of PDMS elements.^32^, ^56^ Advances in microfabrication techniques endow these chips with more elaborate and biomimetic structure designs. However, the acquirement of microfluidic chips is just the first step, while the following function evaluation in a precise manner is more challenging, which brings forward high requirements on the detection modules. It is worth mentioning that the construction of composite systems with multiple organs should also be taken into consideration in the design of microfluidic chips.

In addition to addressing the bottleneck problem at the technical level, future endeavors should be focused on promoting the practical values of organs‐on‐chips. At present, the biologists are mainly depending on these platforms to explore the underlying biological mechanisms for more accurate drug evaluation. Nevertheless, how the organs‐on‐chips adapt to market requirements is still an issue to be addressed. On one hand, the standardized production of organs‐on‐chips should be propelled to make them step out of the laboratory, including but not limited to standardizing their terminology, classification and technical assessment. On the other hand, the researchers can enhance the communication with pharmaceutical enterprises to set up a commercial standard. In fact, there have been various successful cases such as the commercialized multi‐organ models from the collaboration of Institute of Biotechnology at Technische Universität Berlin and TissUse company, the PhysioMimix™ platform co‐developed by MIT and the Defense Advanced Research Projects Agency, etc.^104^ Despite great achievements, their further industrial transformation still requires the establishment of standardized guidelines, regulatory frameworks, and so on. More importantly, when the organs‐on‐chips are magnified for artificial organs in medical applications, the further improvement of their functions is a promising aspect.

In summary, there are still a great deal of controversy and challenges need to be resolved in the field of organs‐on‐chips owing to the lack of deep interdisciplinary cooperation. Whether the replacement of animal experiments or the exploitation of artificial organs, these attempts will not succeed overnight. As an intermediate product, the organs‐on‐chips would play an important role in facilitating this process. Therefore, it is conceivable that organs‐on‐chips would find broad prospects and even drive the development of related fields in the future.

## AUTHOR CONTRIBUTIONS

Yuanjin Zhao conceived the idea; Lingyu Sun wrote the manuscript; Hanxu Chen, Dongyu Xu and Rui Liu revised the manuscript.

## CONFLICT OF INTEREST STATEMENT

The authors declare no competing financial interests.
